# Supplementary material for: The rate of transient beta frequency events predicts behavior across tasks and species
Source: eLife. 2017 Nov 6;6:e29086. doi: 10.7554/eLife.29086 (PMC5683757; doi:10.7554/eLife.29086)
Supplement: Transparent reporting form [file elife-29086-transrepform.docx]

***eLife’s* transparent reporting form**

We encourage authors to provide detailed information *within their submission* to facilitate the interpretation and replication of experiments. If you have any questions, please contact us: [editorial@elifesciences.org](mailto:editorial@elifesciences.org).

**Sample-size estimation**

- You should state whether an appropriate sample size was computed when the study was being designed
- You should state the statistical method of sample size computation and any required assumptions
- If no explicit power analysis was used, you should describe how you decided what sample (replicate) size (number) to use

Please outline where this information can be found within the submission (e.g., page numbers or figure legends), or explain why this information doesn’t apply to your submission:

The human MEG data in this study was collected for a prior study and published previously. For mouse data, we selected the same number of sessions (N=10) as the number of subjects for the two human MEG studies. The mouse detection sessions were chosen based on behavioral performance only, as described in page 10 (Methods, “Behavioral analysis” section). We did not perform explicit power analysis in our current study design.

**Replicates**

- You should report how often each experiment was performed
- You should include a definition of biological versus technical replication
- The data obtained should be provided and sufficient information should be provided to indicate the number of independent biological and/or technical replicates
- If you encountered any outliers, you should describe how these were handled
- Criteria for exclusion/inclusion of data should be clearly stated
- High-throughput sequence data should be uploaded before submission, with a private link for reviewers provided (these are available from both GEO and ArrayExpress)

Please outline where this information can be found within the submission (e.g., page numbers or figure legends), or explain why this information doesn’t apply to your submission:

Information on how often each experiment was performed can be found on page 8 for human MEG data collection, and page 15 for mouse behavior. After preprocessing for artifacts, no outliers were excluded. Details on preprocessing for artifacts can be found in previous publications from our group (Jones et al., 2007 J. Neurosci; Jones et al., 2010 J. Neurosci; Sacchet et al., 2015 J. Neurosci) for the human MEG data, and on page 14 for mouse LFP data (Methods, “Chronic extracellular electrophysiology in mice” section).

**Statistical reporting**

- Statistical analysis methods should be described and justified
- Raw data should be presented in figures whenever informative to do so (typically when N per group is less than 10)
- For each experiment, you should identify the statistical tests used, exact values of N, definitions of center, methods of multiple test correction, and dispersion and precision measures (e.g., mean, median, SD, SEM, confidence intervals; and, for the major substantive results, a measure of effect size (e.g., Pearson's r, Cohen's d)
- Report exact p-values wherever possible alongside the summary statistics and 95% confidence intervals. These should be reported for all key questions and not only when the p-value is less than 0.05.

Statistics used in this paper include: Wilcoxon signed-rank test, paired t-test, Cohen’s d, Friedman test, Pearson’s r, and bootstrapping for calculating confidence interval for ideal observer analysis. Details of the statistical tests along with p-values and summary statistics are described in the text, figures and figure legends.

Please outline where this information can be found within the submission (e.g., page numbers or figure legends), or explain why this information doesn’t apply to your submission:

(For large datasets, or papers with a very large number of statistical tests, you may upload a single table file with tests, Ns, etc., with reference to page numbers in the manuscript.)

**Additional data files (“source data”)**

- We encourage you to upload relevant additional data files, such as numerical data that are represented as a graph in a figure, or as a summary table
- Where provided, these should be in the most useful format, and they can be uploaded as “Source data” files linked to a main figure or table
- Include model definition files including the full list of parameters used
- Include code used for data analysis (e.g., R, MatLab)
- Avoid stating that data files are “available upon request”

Please indicate the figures or tables for which source data files have been provided:

The 1 second prestimulus MEG and LFP traces and their Morlet-convolved time-frequency representations can be found in Matlab formatted matrices (<http://datadryad.org/review?doi=doi:10.5061/dryad.pn931>). Matlab scripts for data analysis is available at <https://github.com/hs13/BetaEvents>.
